# Effect of Position Change From the Bed to a Wheelchair on the Regional Ventilation Distribution Assessed by Electrical Impedance Tomography in Patients With Respiratory Failure

**DOI:** 10.3389/fmed.2021.744958

**Published:** 2021-11-04

**Authors:** Siyi Yuan, Yi Chi, Yun Long, Huaiwu He, Zhanqi Zhao

**Affiliations:** ^1^Department of Critical Care Medicine, State Key Laboratory of Complex Severe and Rare Diseases, Peking Union Medical College Hospital, Chinese Academy of Medical Science and Peking Union Medical College, Beijing, China; ^2^Department of Biomedical Engineering, Fourth Military Medical University, Xi'an, China; ^3^Institute of Technical Medicine, Furtwangen University, Villingen-Schwenningen, Germany

**Keywords:** electrical impedance tomography (EIT), regional lung ventilation, body position change, early mobilizations, bedside monitoring

## Abstract

**Background:** There is limited knowledge about the effect of position change on regional lung ventilation in patients with respiratory failure. This study aimed to examine the physiological alteration of regional lung ventilation during the position change from lying in bed to sitting on a wheelchair.

**Methods:** In this study, 41 patients with respiratory failure who were weaned from the ventilators were prospectively enrolled. The electrical impedance tomography (EIT) was used to assess the regional lung ventilation distribution at four time points (T_base_: baseline, supine position in the bed, T_30min_: sitting position in the wheelchair after 30 min, T_60min_: sitting position in the wheelchair after 60 min, T_return_: the same supine position in the bed after position changing). The EIT-based global inhomogeneity (GI) and center of ventilation (CoV) indices were calculated. The EIT images were equally divided into four ventral-to-dorsal horizontal regions of interest (ROIs 1–4). Depending on the improvement in ventilation distribution in the dependent regions at T_60min_ (threshold set to 15%), the patients were divided into the dorsal ventilation improved (DVI) and not improved (non-DVI) groups.

**Results:** When the patients moved from the bed to a wheelchair, there was a significant and continuous increase in ventilation in the dorsal regions (ROI 3 + 4: 45.9 ± 12.1, 48.7 ± 11.6, 49.9 ± 12.6, 48.8 ± 10.6 for T_base_, T_30min_, T_60min_, and T_return_, respectively; *p* = 0.015) and CoV (48.2 ± 10.1, 50.1 ± 9.2, 50.5 ± 9.6, and 49.5 ± 8.6, *p* = 0.047). In addition, there was a significant decrease in GI at T_60min_ compared with T_base_. The DVI group (*n* = 18) had significantly higher oxygenation levels than the non-DVI group (*n* = 23) after position changing. ROI4_Tbase_ was significantly negatively correlated with the ΔSpO_2_ (R = 0.72, *p* < 0.001). Using a cutoff value of 6.5%, ROI4_Tbase_ had 79.2% specificity and 58.8% sensitivity in indicating the increase in the dorsal region related to the position change. The corresponding area under the curve (AUC) was 0.806 (95% *CI*, 0.677–0.936).

**Conclusions:** Position change may improve the ventilation distribution in the study patients. The EIT can visualize real-time changes of the regional lung ventilation at the bedside to guide the body position change of the patients in the intensive care unit (ICU) and measure the effect of clinical practice.

**Trial Registration:** Effect of Early Mobilization on Regional Lung Ventilation Assessed by EIT, NCT04081129. Registered 9 June 2019—Retrospectively registered. https://register.clinicaltrials.gov/prs/app/action/SelectProtocol?sid=S00096WT&selectaction=Edit&uid=U00020D9&ts=2&cx=v2cwij.

## Background

It is recommended that the patients in the intensive care unit (ICU) receive protocolized rehabilitation and early mobilization (1). As one method of early mobilization, the position changes from lying in bed to sitting on a wheelchair are commonly carried out in the ICU (2). Some studies have shown that body position changes could affect lung ventilation (3–5). However, another study reported that lateral body position does not yield ventilation changes (6). Considering the manpower required for body position change, it is controversial whether all the patients in the ICU need to carry out position change and mobilization. Bedside identification of the patients who would benefit from the position change is warranted. In addition to improving strength and functional status, promoting the recovery of lung function is also one of the main purposes of body position changes. On the one hand, the body position changes influence the movement of the diaphragm (7). On the other hand, it is still not clear whether the position change from the bed to a wheelchair can truly promote lung ventilation distribution.

The electrical impedance tomography (EIT) is a non-invasive, non-ionizing monitoring system that can obtain real-time images of regional lung ventilation at the bedside by monitoring the electrical impedance changes in the underlying tissue (8, 9). EIT has already been used for positive end-expiratory pressure (PEEP) titration in patients with acute respiratory distress syndrome (ARDS) (10, 11) and position changes (12). Little data are available investigating the changes in regional lung ventilation during position change from lying in bed to sitting on a wheelchair. This study aimed to examine whether EIT can be used to determine the impact of position change on the regional ventilation distribution and to identify the patients who would benefit from this process.

## Patients and Methods

### Patients

This study involving human subjects was approved by the Institutional Research and Ethics Committee of the Peking Union Medical College Hospital (JS-1,896), Beijing, China. The written informed consent was obtained from the next of kin of the patient before enrollment.

Adult critically ill patients with respiratory failure were enrolled in this study. The inclusion criteria were as follows: (1) the patients with a respiratory failure-partial pressure of oxygen/fraction of inspiration oxygen (PaO_2_/FiO_2_) <300, oxygen saturation (SpO_2_) <92%, or respiration rate (RR) > 25 bpm at ICU admission; (2) the patients who had a failure in spontaneous breathing trials (SBTs) or had mechanical ventilation duration longer than 1 week; and (3) the patients with conditions requiring position change from bed to a wheelchair (e.g., ICU duration ≥1 week and high risk of respiratory complications). The patients were excluded from the study if they were aged <18 years, were pregnant, had a body mass index (BMI) higher than 50 kg/m^2^, had a ribcage malformation, or had any contraindications for EIT monitoring (automatic implantable cardioverter defibrillator, chest skin injury, etc.), or had any contraindications for position change (hemodynamic instability, FiO_2_ > 60%, arrhythmia, etc.).

### Physiological Measurements

We collected baseline data on the day of EIT measurement, such as age, sex, tracheotomy, ICU duration, days of mechanical ventilation, failed SBT, weaning duration, and type of surgery. The PaO_2_/FiO_2_ and PaCO_2_ were measured at ICU admission. The FiO_2_ was measured at the start of the EIT measurement and remained the same during the intervention. The respiratory parameters, such as RR, SpO_2_, and hemodynamic parameters, such as heart rate (HR) and mean arterial pressure (MAP), were obtained at different time points.

### Experimental Protocol

The participants went through the body position change from lying in bed to sitting position in a wheelchair. The condition of a patient before the position change was defined as the baseline condition in a supine position on the bed. Then, the patients were moved from the bed to a wheelchair and sat for 1 h. Afterward, the patients returned to the supine position on the bed. During this process, the patients had an EIT belt fixed on their chest to monitor their regional lung ventilation at four time points (T_base_: baseline, supine position before position change, T_30min_: sitting position in the wheelchair for 30 min, T_60min_: sitting position in the wheelchair for 60 min, T_return_: the same supine position on the bed after the position change). Moreover, the patients spontaneously breathed with a fixed FiO_2_ level during the process.

### EIT Measurements

The EIT measurements were performed with PulmoVista500 (Dräger Medical, Lübeck, Germany). During the protocol, a silicone EIT belt with 16 electrodes was attached on the surface of the chest of the patient in one transverse plane corresponding to the fourth intercostal parasternal space and was then connected to the EIT monitor for bedside visualization (13). When the patient moved to different positions, we ensured that the 16-electrode array was attached at the same level on the chest surface so that the lung ventilation results were comparable. The electrical alternating currents were applied in a sequential rotating process through the adjacent electrode pairs. The resulting differences in surface potential between the neighboring electrode pairs were measured (14). The stimulation frequency and amplitude were adjusted automatically by the EIT device to minimize the influence of background noise. The EIT measurements were continuously sampled at 20 Hz. In addition, the data were digitally filtered using a low-pass filter with a cutoff frequency of 0.67 Hz to eliminate the cardiac-related impedance changes. The EIT data were analyzed offline by a computer program.

### Analysis of the EIT Data

The EIT data spanning 2 min were acquired at baseline and at different time points. The tidal images were defined as the different images between the end-inspiration and end-expiration. The tidal images for an average of 2 min were calculated for each position to minimize the influence of spontaneous breathing. The mean tidal images were divided into four symmetrical, non-overlapping ventral-to-dorsal horizontal regions of interest (ROIs), ranging from the gravity-independent area to the gravity-dependent area, namely, the ventral (ROI1), midventral (ROI2), middorsal (ROI3), and dorsal (ROI4) regions.

The global inhomogeneity (GI) index can indicate the overall change in the inhomogeneity of ventilation and the local lung distribution status (15). In brief, the sum of the differences between the individual pixels and the average value was calculated. This summed value was normalized to the amplitude of impedance tidal variation. A lower GI index value indicated higher ventilation homogeneity.

The center of ventilation (CoV) describes the weighted geometrical center of the ventilation distribution (16–18). When the tidal ventilation region is mainly distributed in the dependent lung region, the CoV value is larger.

The patients were divided into two groups: (1) dorsal ventilation improved group (DVI group): ventilation distribution of ΔROI (3 + 4)/ROI (3 + 4)_Tbase_ ≥ 15% and (2) dorsal ventilation not improved group (non-DVI group): ventilation distribution ΔROI (3 + 4)/ ROI (3 + 4)_Tbaseline_ <15%, [Δ ROI (3 + 4) = ROI (3 + 4)_T60min_− ROI (3 + 4)_Tbase_].

### Statistical Analysis

This is a self-controlled paired study, and the sample size was calculated using the following formula:


$$\begin{array}{l} n={\left(Z_{1}-\frac{a}{2}+Z_{1}-\beta \right)}^{2}Sd^{2}/{\left(\mu t-\mu c\right)}^{2} \end{array}$$

where α is 0.05, β is 0.1, and SD is the standard deviation of the difference before and after the intervention. μt–μc is the difference between the follow-up and baseline groups. The sample size was estimated based on ROI4 (ventilation in the dorsal region). The μt (SD) and μc (SD) were 4.1 (0.98) and 5.2 (1.87) based on a previous study (19), respectively, with a correlation coefficient of 0.6. The minimum sample size to obtain a study power of 90% was 29 pairs.

The normally distributed results were presented as mean ± SD whereas non-normally distributed results were presented as median (25th−75th percentile). The paired data at different time points were compared with the paired sample *T*-test or Wilcoxon's signed rank test depending on the data distribution. Mann–Whitney test was used to compare the groups on continuous variables, and chi-square and Fisher's exact tests were used to compare the categorical variables. The comparisons of two continuous variables were performed using Spearman's correlation. Trend comparisons of the related parameters on different days were performed using a General Linear Model Repeated Measures, or so-called repeated measure ANOVA (RM-ANOVA) (20). This RM-ANOVA model is an extension of the classical ANOVA, which allows handling both the fixed effect (different days) and random effect (patient). Bonferroni correction was used to adjust the *p*-value for multiple comparisons. The best cut-off value from a ROC curve was chosen to reach the highest Youden's index. The statistical analysis was performed with SPSS 23.0 (IBM, Armonk, NY, USA) and Prism 7 (GraphPad Software, San Diego, CA, USA). A *p*-value smaller than 0.05 was considered statistically significant.

## Results

From January 2019 to January 2020, this study enrolled 41 patients. The main characteristics of the patients are summarized in Table 1. The mean patient age was 64.3 ± 14.1 years, and 33/41 (80%) patients were men. Nine of 41 (22%) patients underwent a tracheotomy. The mean ICU duration was 14.3 ± 13.3 days.

**Table 1:** Main characteristics of the study population.

**Characteristics**	**Data**
Age, y	64.3 ± 14.1
Male, *n* (%)	33 (80.5)
BMI, kg/m^2^	21.3 ± 3.7
APACHE-II	11.5 ± 3.6
PaO_2_/FiO_2_, mmHg	247.3 ± 43.5
PaCO_2_, mmHg	40.2 ± 5.7
FiO_2_	0.33 ± 0.05
Cardiosurgery, *n* (%)	12 (29.2)
Preexisting COPD, *n* (%)	3 (7.3)
Septic shock, *n* (%)	15 (36.6)
ARDS, *n* (%)	11 (26.8)
Days of intubation	7.8 ± 5.3
ICU length of stay, days	14.3 ± 13.3

*ARDS, Acute respiratory distress syndrome; COPD, Chronic Obstructive Pulmonary Disease.*

*Data are given as mean ± SD*.

### Effects of Position Change on Regional Lung Ventilation Distribution

**Figure 1:**
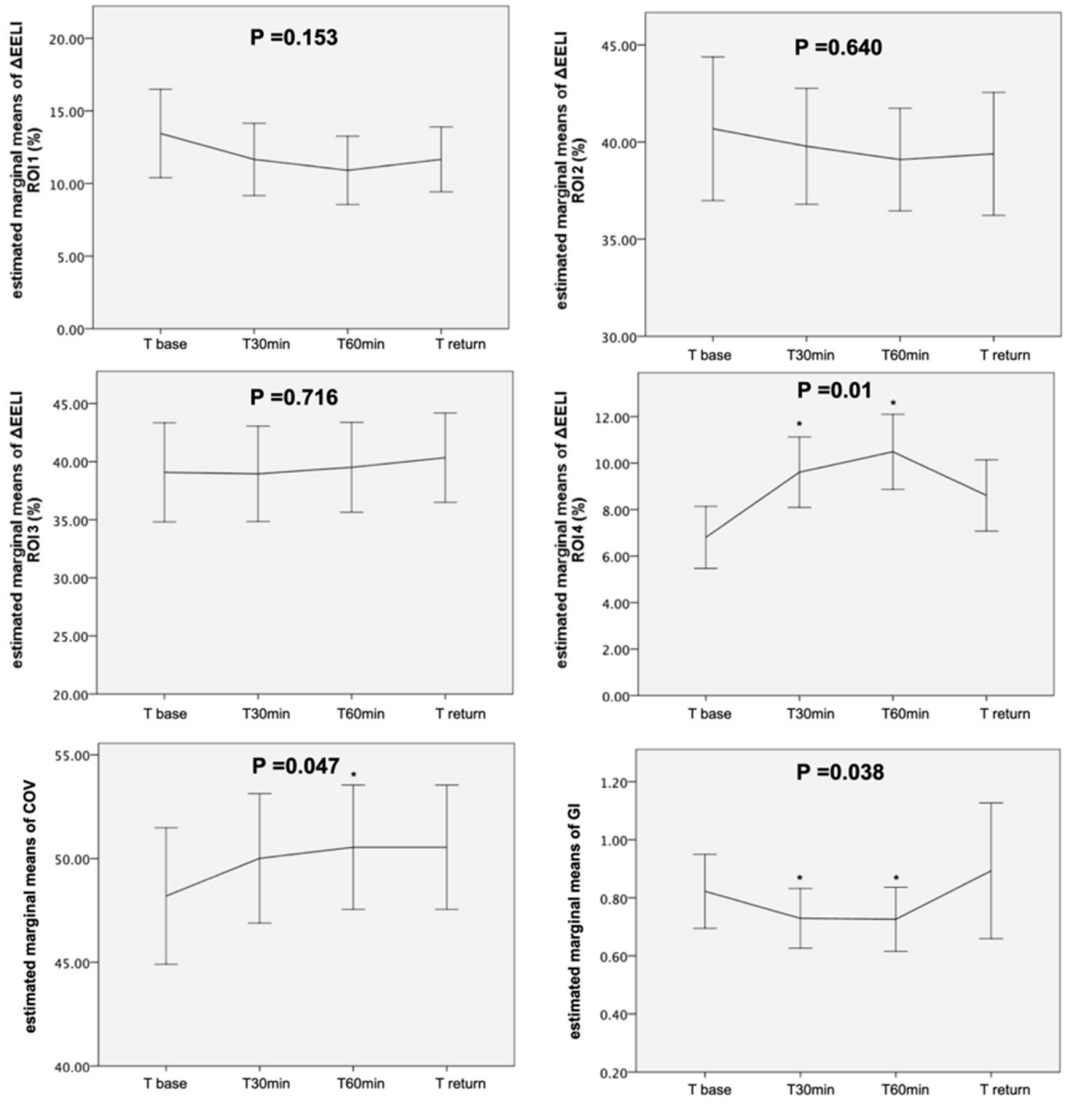
Evolution of the estimated marginal means of ROI 1–4, GI, and COV at different time points. ROI, region of interest; GI, global inhomogeneity; COV, center of ventilation; *p* value by general linear model repeated measures. ^*^p < 0.05 indicates a significant difference between this time point and T_base_.

The ROI4 (most dorsal region) trend significantly increased (*p* = 0.01) after a position change, but ROIs 1–3 showed no significant differences across the study time points. Moreover, GI significantly decreased (*p* = 0.038), and CoV significantly increased (*p* = 0.047) from the baseline to T_60min_ (Figure 1).

### Effects of Position Change on the Respiratory and Hemodynamic Parameters

For the respiratory parameters, a significant increase was observed in SpO_2_ (*p* = 0.045) and RR (*p* = 0.001). In addition, the heart rate significantly increased after the position change (*p* = 0.011). However, MAP remained stable across all the study time points (Table 2).

**Table 2:** Changes of respiratory and hemodynamics parameters at different time points.

	**T_**base**_**	**T_**30min**_**	**T_**60min**_**	**T_**return**_**	**Trend-*p***
HR, bpm	85.5 ± 11.6	89.3 ± 14.9*	90.2 ± 14.6*	87.2 ± 12.5	0.011
MAP, mmHg	82.4 ± 11.3	82.4 ± 11.9	84.5 ± 11.7	84.0 ± 12.0	0.491
SpO_2_, %	90.6 ± 2.7	91.8 ± 1.9	92.9 ± 1.6*	91.0 ± 2.3	0.045
RR, /min	19.5 ± 4.8	22.3 ± 5.2*	22.7 ± 5.7*	21.0 ± 4.8*	0.001

HR, Heart rate; MAP, Mean arterial pressure; SpO_2_, Peripheral oxygen saturation; RR, Respiratory rate; bpm, beasts per minute. T_base_, supine position baseline; T_30min_, sitting position for 30min; T_60min_, sitting position for 60min;T_return_, return to supine position from sitting position. p value by General Linear Model Repeated Measures. Data are given as mean ± SD. ^*^p < 0.05 by post hoc, indicates the significantly difference between this timepoint and T_base_.

### Relationships Between ΔROI 3 + 4 and ΔSpO_2_, ROI 4_(Base)_ and ΔROI 3 + 4, and ROI 4_(Base)_ and ΔSpO_2_

**Figure 2:**
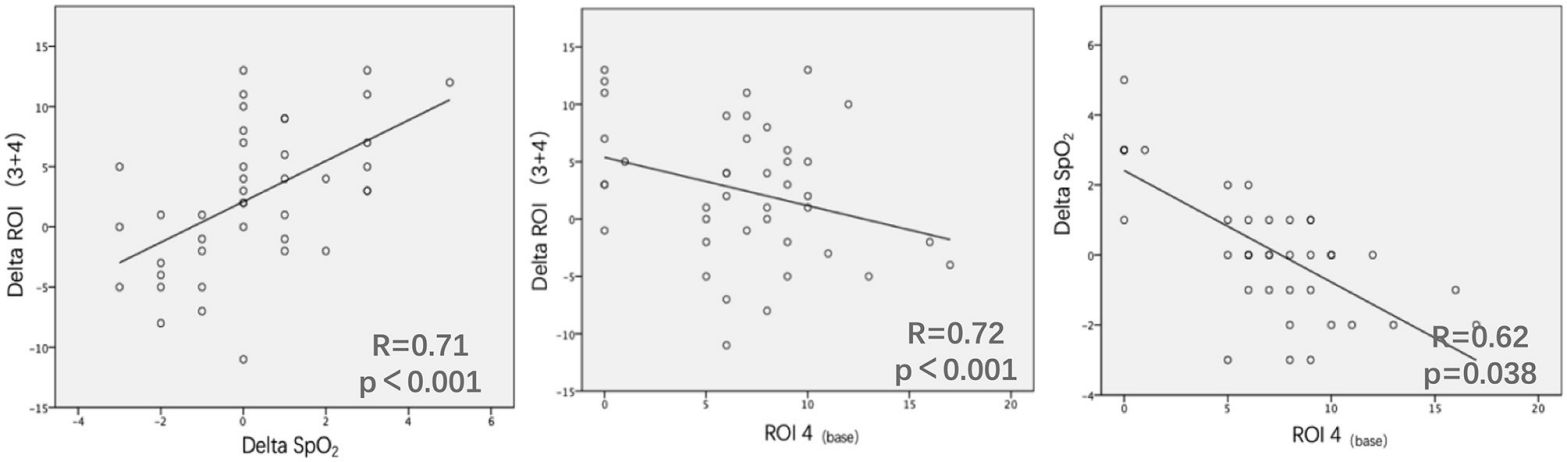
Relationship between ΔROI 3 + 4 and ΔSpO_2_, ROI 4_(base)_ and ΔROI 3 + 4, and ROI 4_(base)_ and ΔSpO_2_ in 41 patients. ΔROI 3 + 4 means ROI 3 + 4 at T_60min_ −ROI 3 + 4 at T_base_. ΔSpO_2_ means SpO_2_ at T60 min–SpO_2_ at T_base_. ROI 4(base) means ROI 4 at T_base_. Some data points overlap.

Regarding the changes, we found that ROI (3 + 4) at T_60min_ − ROI (3 + 4) at T_base_ [ΔROI (3 + 4)] and SpO_2_ at T_60min_ − SpO_2_ at T_base_ (ΔSpO_2_) were positively related (*R* = 0.71, *p* < 0.001). Moreover, ROI 4 at T_base_ [ROI 4_(base)_] was negatively related to ΔSpO_2_ (*R* = 0.72, *p* < 0.001) and ΔROI (3 + 4) (*R* = 0.62, *p* = 0.038) (Figure 2).

### Differences Between the DVI and Non-DVI Groups

In the DVI and non-DVI groups, 18 and 23 patients were included, respectively. A comparison of the baseline characteristics of the two groups is shown in Table 3. There were no significant differences in age, PaO_2_/FiO_2_, HR, RR, or ICU duration (days) between these two groups at baseline. The DVI group had a longer duration of mechanical ventilation (9.9 ± 5.2 vs. 6.1 ± 4.8, *p* = 0.018) and a higher incidence of SBT failure than the non-DVI group (61.1 vs. 30.4%, DVI and non-DVI groups, respectively; *p* = 0.048).

**Table 3:** Comparison baseline date in DVI group and Non-DVI group.

**Variables**	**DVI group**	**Non-DVI**	***p*-value**
	***N* = 18**	***N* = 23**	
Age, y	63.8 ± 13.2	64.5 ± 14.3	0.481
Male, *n* (%)	14 (77.8)	19 (82.6)	0.709
BMI, kg/m^2^	21.2 ± 4.3	22.0 ± 3.8	0.567
APACHE-II	11.8 ± 3.2	11.2 ± 4.5	0.630
ICU length of stay, days	15.3 ± 4.2	13.8 ± 2.1	0.345
Tracheotomy, *n* (%)	6 (33.3)	3 (13.0)	0.056
MV duration, days	9.9 ± 5.2	6.1 ± 4.8	0.018*
SBT failure, *n* (%)	11 (61.1)	7 (30.4)	0.048*
Weaning duration	1.7 ± 1.4	1.3 ± 0.5	0.189
PaO_2_/FiO_2_, mmHg	233.5 ± 45.1	256.2 ± 56.2	0.162
PaCO_2_, mmHg	39.8 ± 7.2	40.1 ± 4.5	0.515
ROI 1	14.2 ± 11.6	12.8 ± 8.0	0.833
ROI 2	43.0 ± 11.7	38.9 ± 11.7	0.212
ROI 3	36.9 ± 11.7	40.8 ± 14.8	0.408
ROI 4	5.9 ± 4.0	7.5 ± 4.4	0.043*
HR, bpm	83.2 ± 8.4	87.2 ± 13.4	0.124
MAP, mmHg	80.8 ± 11.3	83.3 ± 11.2	0.782
RR, min^−1^	19.7 ± 4.1	19.2 ± 5.2	0.620

MV, mechanical ventilation; SBT, spontaneous breathing trials; HR, Heart rate; MAP, mean arterial pressure; SpO_2_, peripheral oxygen saturation; RR, respiratory rate; bpm, beasts per minute. DVI group, dorsal-ventilation improved group; Non-DVI group, dorsal-ventilation not-improved group. Data are given as mean ± SD. *P < 0.05.

**Figure 3:**
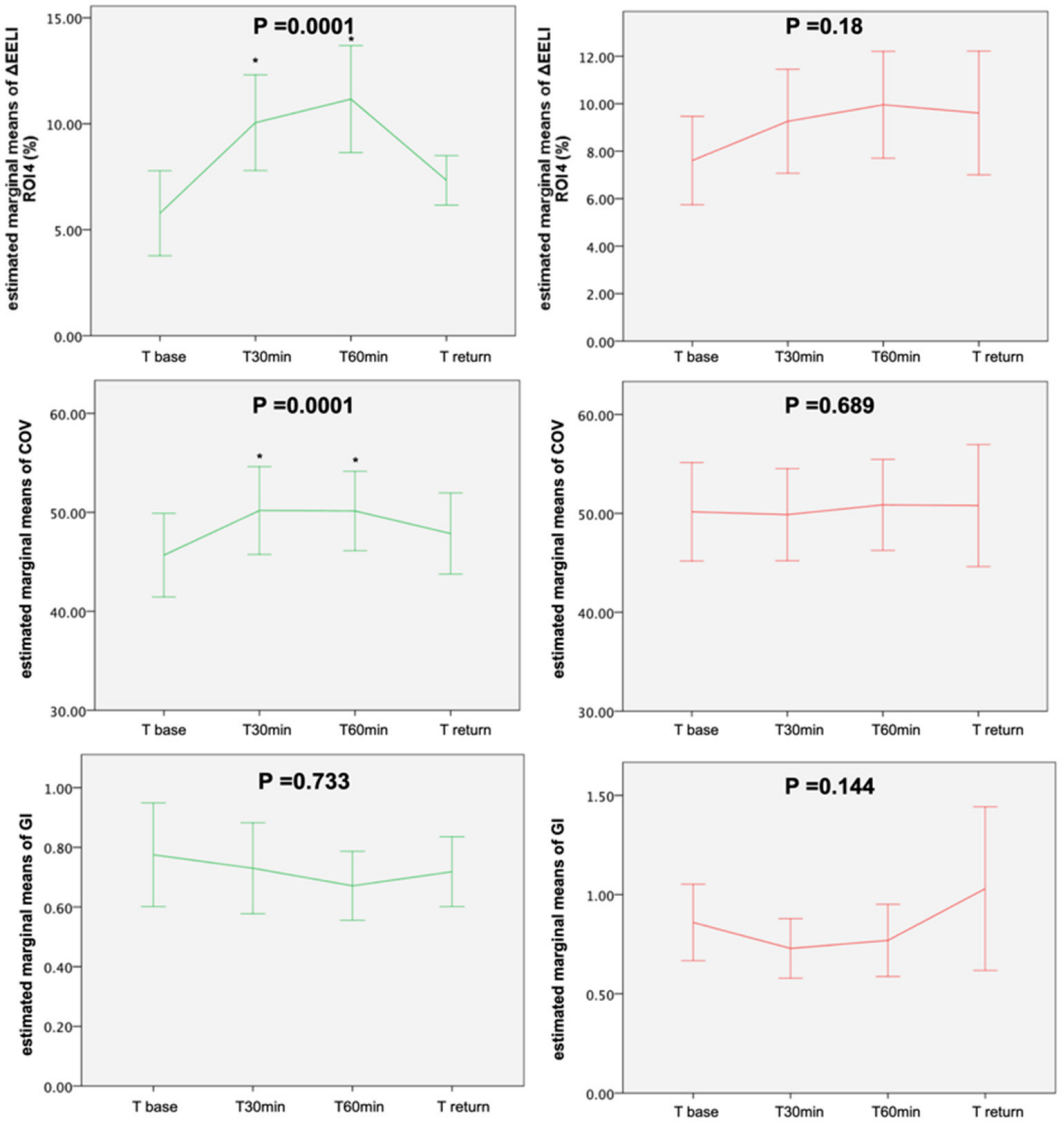
Evolution of the estimated marginal means of ROI 1–4, GI and COV at different time points in two subgroups. GI, global inhomogeneity; COV, center of ventilation; DVI, dorsal-ventilation improved group; Non-DVI, dorsal-ventilation not-improved group. The green line indicates the DVI group and the red line indicates the non-DVI group. *p* value by general linear model repeated measures. *p < 0.05 indicates a significant difference between this time point and T_base_.

The changes in the respiratory and hemodynamic parameters in these two groups across the study time points are shown in Table 4. The changes in ROI 4, GI, and CoV across time points are shown in Figure 3. The ROI 4 (*p* = 0.0001) and COV (*p* = 0.0001) significantly increased from the baseline to T_60min_ in the DVI group but did not significantly increase in the non-DVI group. In addition, HR significantly increased after body position change in the non-DVI group (*p* < 0.0001), but not in the DVI group (*p* < 0.342). And there was a significant increase of RR both in the non-DVI (*p* < 0.0001) and DVI groups (*p* = 0.011) after position change.

**Table 4:** Subgroup analysis of changes of respiratory and hemodynamics parameters at different time points.

		**T_**base**_**	**T_**30min**_**	**T_**60min**_**	**T_**return**_**	**Trend-*p***
SpO_2_, %	DVI	90.8 ± 3.1	92.6 ± 2.1	93.2 ± 1.3*	90.4 ± 2.5	0.035
	Non-DVI	92.1 ± 2.3	91.1 ± 1.9	92.2 ± 1.3	92.3 ± 2.2	0.567
MAP, mmHg	DVI	80.8 ± 11.3	79.5 ± 7.8	82.5 ± 9.3	80.6 ± 11.7	0.508
	Non-DVI	83.3 ± 11.2	84.4 ± 16.9	85.8 ± 13.1	86.1 ± 11.8	0.663
HR, bpm	DVI	83.2 ± 8.4	83.7 ± 8.1	84.3 ± 8.2	82.7 ± 7.6	0.342
	Non-DVI	87.2 ± 13.4	90.4 ± 17.3*	94.6 ± 16.7*	90.6 ± 14.2	<0.0001
RR, min^−1^	DVI	19.7 ± 4.1	21.4 ± 4.6	21.7 ± 4.9*	21.1 ± 4.8	0.011
	Non-DVI	19.2 ± 5.2	22.7 ± 5.7*	23.2 ± 6.3*	20.7 ± 4.9	<0.0001

HR, Heart rate; MAP, Mean arterial pressure; SpO_2_, Peripheral oxygen saturation; DVI, dorsal-ventilation improved group; Non-DVI, dorsal-ventilation not-improved group; T_base_, supine position baseline; T_30min_, sitting position for 30min; T_60min_, sitting position for 60min;T_return_, return to supine position from sitting position; p value by General Linear Model Repeated Measures. Data are given as mean ± SD. *p < 0.05 by post hoc, indicates the significantly difference between this timepoint and T_base_.

### Using ROI 4_(Base)_ to Distinguish the DVI Patients

**Figure 4:**
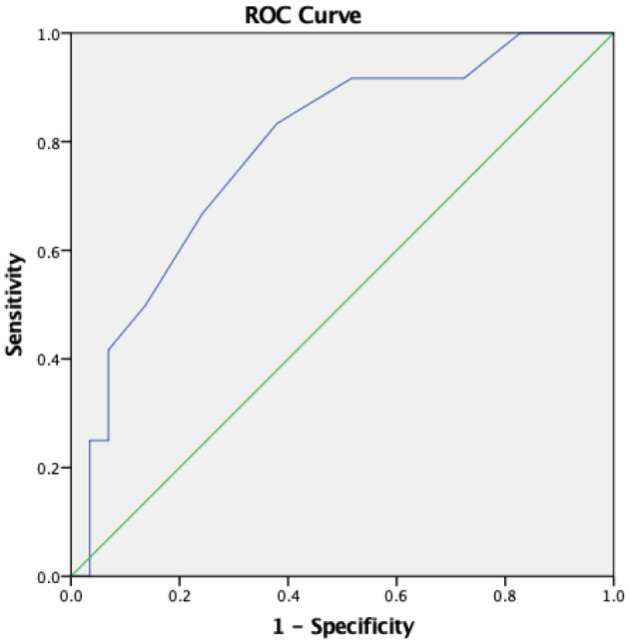
ROC curve using ROI 4_(*base*)_ to distinguish DVI patients (AUC 0.806).

If we used ROI 4 at baseline to distinguish the DVI group from the non-DVI group, the ROC curve of the diagnostic value of ROI 4_(base)_ is shown in Figure 4. The best cutoff for ROI 4_(base)_ is 6.5%, which means that in patients with an initial ROI of 4_(base)_ <6.5%, a position change to sitting on a wheelchair may improve the oxygenation and likely should be performed. The specificity was 79.2%, while the sensitivity was only 58.8%. The area under the curve (AUC) was 0.806 (95% *CI*, 0.677–0.936).

## Discussion

**Figure 5:**
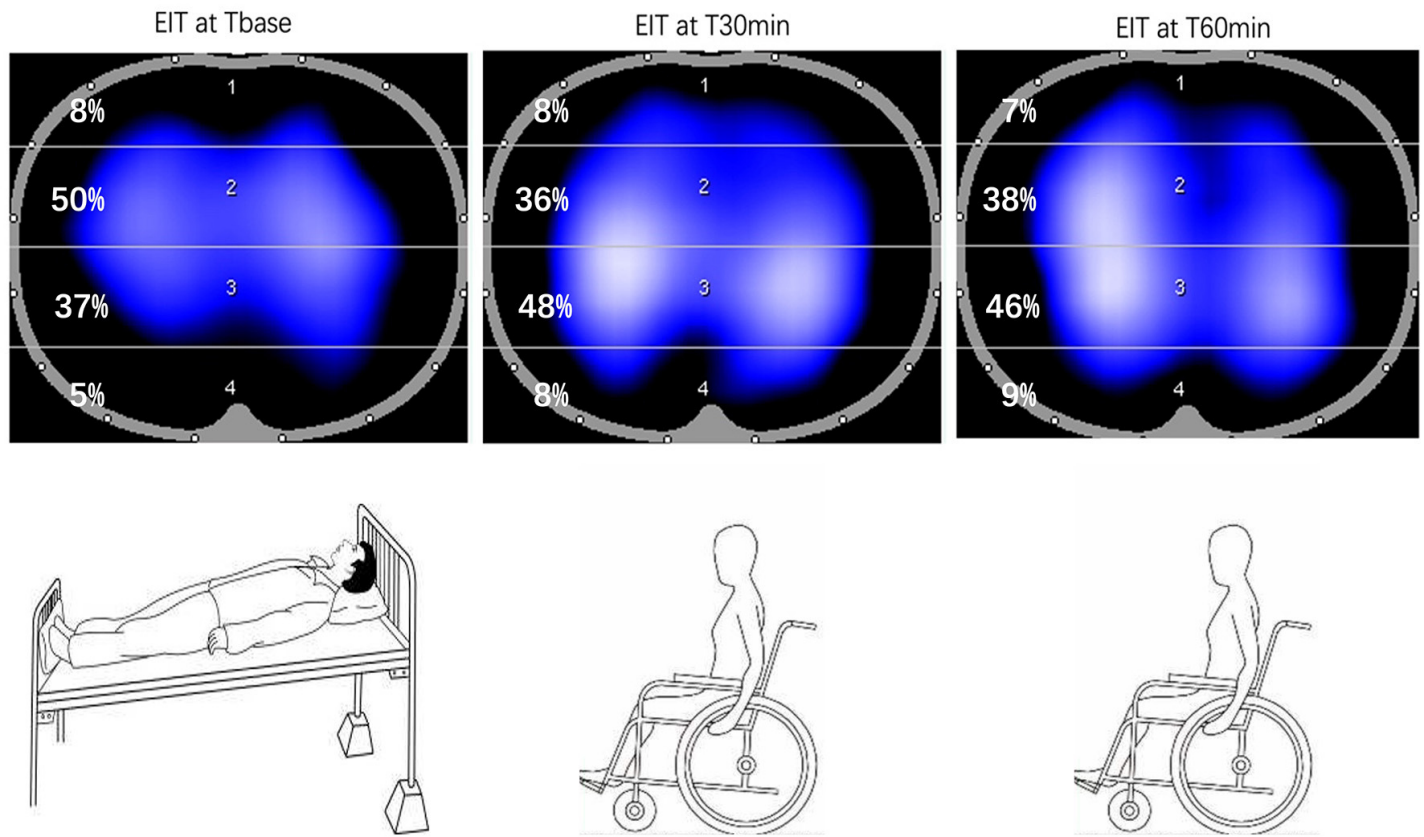
EIT image from supine position (T_base_) to sitting position on a wheelchair. T_base_: baseline, supine position in the bed, T30min: sitting position in the wheelchair after 30 min, T60min: sitting position in the wheelchair after 60 min.

In this study, we focused on the body position change from lying in bed to sitting in a wheelchair. We found that (1) the position change can induce an increase in ventilation in the dorsal region in some but not all patients. (2) EIT is feasible for monitoring the regional ventilation distribution changes during the position change and the EIT image is shown in Figure 5. (3) ROI 4_(Tbase)_ is negatively related with ΔSpO_2_ and lung ventilation in the dorsal regions, which suggests that the baseline ventilation distribution in the most dorsal regions might indicate the ventilation changes due to the position changes.

### Effects of Body Position Change on Lung Ventilation

As shown in the previous studies, early mobilization may induce changes in lung ventilation, thereby preventing postoperative respiratory complications and decreasing the number of days a patient requires ventilation (21–23). However, the results shown in other studies are inconsistent (24, 25). A previous study showed that the body position could affect lung ventilation (3). Another study also reported that the sitting position is associated with the improvement of functional residual capacity and oxygenation and reduction of work of breathing (26). In our study, the body position change-induced lung ventilation in dorsal lung areas in some but not in all patients, which explains the inconsistent results shown in the previous studies. We acknowledge that sitting on a bed and sitting on a wheelchair may have different effects on lung ventilation due to additional transportation, which was not explored in the current study.

Furthermore, we divided all the patients into two groups: (a) the patients in the DVI group showed increased ventilation in the dorsal region and better oxygenation after a position change. (b) The patients in the non-DVI group did not have increased dorsal ventilation from this process, and there was no difference in the oxygenation or ventilation. Thus, the patients classified as type DVI may have a greater potential for increased ventilation, which indicates that sitting on a wheelchair may benefit these patients and is recommended. The patients classified as type non-DVI may have limited potential for improved ventilation, and the mobilization should be introduced cautiously. In addition, the results showed that the DVI group had a longer duration of mechanical ventilation and a higher incidence of SBT failure than the non-DVI group, which means that patients in the DVI group were more likely to suffer from diaphragm weakness. The diaphragm weakness may play a role in the difference between the two groups. *We also found out that non-DVI had a significant increase in HR and RR after a body position change. Interestingly, the disturbed HR and RR caused by position change were less prominent in the DVI group. Because oxygenation improvement and regional lung recruitment were limited during position change, a higher increase of HR and RR in the non-DVI group would be compensation for the increase of oxygen demand. On the contrary, the patients in the DVI group had a better improvement of regional ventilation and oxygenation, so that a less change of HR and RR caused by the position change was found*.

### EIT on Regional Lung Ventilation

Although EIT is non-invasive, it takes time to perform the measurements and requires proper training, such as the interpretation of data. Available as a bedside tool, ultrasound may provide a fast assessment of lung status, e.g., consolidation and pleural effusion. However, the results of ultrasound examination may be operator-dependent and cannot be used for continuous monitoring. A potential benefit of EIT for ventilation monitoring over days was previously reported (27).

In this study, we used EIT to measure the regional lung ventilation during the position changes from the supine position to sitting on a wheelchair. This intervention influences regional lung ventilation by changing the body position. The previous studies have used EIT to monitor the ventilation improvement during the body position changes (4–6, 28, 29) and have proven the feasibility of EIT. Hough et al. (4) confirmed that body position changes might influence the regional ventilation distribution in infants, whereas van der Burg et al. (5) did not find any differences between the lateral and supine positions. Another study (28) compared EIT measurements in the supine and prone positions found that ventilation in the dependent lung region increased in the prone position.

In this study, the results show that the ROI4, CoV, and GI results were better in the sitting position, which means that more gas entered the dorsal region and the ventilation distribution became more homogenous. This result is in accord with the result in a previous study (30, 31). One study showed that FEV1 increased when the healthy volunteers changed from a supine to a sitting position (30). Another study showed that the sitting position constantly and significantly relieved the expiratory flow limitation and auto-positive end-expiratory pressure, resulting in a dramatic drop in the alveolar pressures in critically ill obese patients under mechanical ventilation (31). Due to the change in the direction of gravity and the shift in the position of the diaphragm, it is possible that the EIT measurements reflected slightly different lung tissues in the supine and sitting positions.

### ROI 4_(Base)_, SpO2, and Lung Ventilation

In the present study, we found SpO_2_ improvements in 78% of the patients in the DVI group (14/18) and in only 26% of the patients in the non-DVI group (6/23). In addition, we found that ΔSpO_2_ and ΔROI (3 + 4) were positively related and ROI 4_(base)_ was negatively related to ΔSpO_2_, which means that the patients with a lower baseline ROI 4 proportion may have a higher possibility of increased ventilation in the dorsal area and improved oxygenation after a position change. In clinical practice, due to limited strength, it is not easy for the patients in ICU to move to a sitting position. EIT may be useful to determine the impact of position change on the regional ventilation distribution and to identify the patients who would benefit from this process (Figure 3).

## Limitations

This study also has some limitations. First, this study enrolled critically ill patients who had respiratory failure before and had already been weaned from mechanical ventilation. We did not exclude severe chronic obstructive pulmonary disease (COPD) or cardiogenic edema patients, so this study may carry certain heterogeneity. Second, we monitored position change only once for 1 h, and the study period may not be long enough to assess the regional ventilation changes. Third, the sample size might not be large enough for the subgroup analysis. Fourth, at the four time points, the patients needed to be moved from the supine position from the bed to the sitting position on the wheelchair. The electrode belt of EIT might have shifted slightly so that a comparison of end-expiratory lung impedance would not be reliable. Therefore, the change in end-expiratory lung volume was not evaluated. Fifth, the threshold used to distinguish the DVI and non-DVI groups (15%) was arbitrary, and its clinical value needs to be studied further. Sixth, we speculated that the diaphragm weakness might play a role in the difference between the DVI and non-DVI groups, but we have not recorded diaphragm data by ultrasound. Future research could include the diaphragm assessment to further explore the mechanism of regional lung ventilation change during the position change. Finally, we have only investigated the effects of body position change from lying in a bed to sitting on a wheel, and the effects of other mobilizations, such as sitting or taking respiratory physiotherapy in bed, should be investigated in the future.

## Conclusion

A position change from the supine position in the bed to the sitting position on a wheelchair can induce increased ventilation in the dorsal lung regions and improve oxygenation in some but not all patients. The baseline ventilation distribution in the most dorsal regions might indicate lung ventilation changes due to the position changes. EIT can visualize real-time changes of regional lung ventilation at the bedside to guide the body position change and to measure the effect of clinical practice.

## Data Availability Statement

The datasets presented in this article are not readily available because of patients' privacy. Requests to access the datasets should be directed to tjmuhhw@163.com.

## Ethics Statement

The studies involving human participants were reviewed and approved by the Institutional Research and Ethics Committee of the Peking Union Medical College Hospital. The patients/participants provided their written informed consent to participate in this study.

## Author Contributions

SY and HH: data curation and writing—original draft. YL: supervision. YC: data curation and software. ZZ: writing—reviewing, editing, and software. All authors contributed to the article and approved the submitted version.

## Funding

The Capital's Funds for Health Improvement and Research (No. 2020-2-40111), the Medical and Health Science and Technology Innovation Project of the Chinese Academy of Medical Sciences (No. 2019-12M-1-001), the Chinese Academy of Medical Sciences (CAMS) Innovation Fund for Medical Sciences (CIFMS) from the Chinese Academy of Medical Sciences (No. 2020-I2M-2-005), the National Key Research and Development Program of China (No. 2020YFC0841300), and the Excellence Program of Key Clinical Specialty of Beijing in 2020. The Beijing Municipal Science and Technology Commission (grant No. Z201100005520051).

## Conflict of Interest

ZZ receives consultant fees from Draeger Medical. The remaining authors declare that the research was conducted in the absence of any commercial or financial relationships that could be construed as a potential conflict of interest.

## Publisher's Note

All claims expressed in this article are solely those of the authors and do not necessarily represent those of their affiliated organizations, or those of the publisher, the editors and the reviewers. Any product that may be evaluated in this article, or claim that may be made by its manufacturer, is not guaranteed or endorsed by the publisher.

## Abbreviations

EIT: electrical impedance tomography
T_base_: baseline, supine position in the bed
T_30min_: sitting position in the wheelchair after 30 min
T_60min_: sitting position in the wheelchair after 60 min
T_return_: the same supine position in the bed after position change
GI: global inhomogeneity
CoV: center of ventilation
ROI: region of interest
DVI: dorsal ventilation improved group
Non-DVI: dorsal ventilation not improved group
AUC: area under the curve
PEEP: positive end-expiratory pressure
ARDS: acute respiratory distress syndrome
PaO_2_/FiO_2_: partial pressure of oxygen/fraction of inspired oxygen
SpO_2_: oxygen saturation
RR: respiration rate
BMI: body mass index
HR: heart rate
MAP: mean arterial pressure.
